# Novel combination of sorafenib and biochanin-A synergistically enhances the anti-proliferative and pro-apoptotic effects on hepatocellular carcinoma cells

**DOI:** 10.1038/srep30717

**Published:** 2016-07-29

**Authors:** Mohieldin M. Youssef, Mai F. Tolba, Noha N. Badawy, Andrew W. Liu, Eman El-Ahwany, Amani E. Khalifa, Suher Zada, Ashraf B. Abdel-Naim

**Affiliations:** 1 The American University in Cairo, New Cairo, 11835 Egypt; 2 Okinawa Institute of Science and Technology Graduate University, OIST, Okinawa, 904-0495 Japan; 3 Pharmacology and Toxicology Department, Faculty of Pharmacy, Ain-Shams University, Cairo, 11566 Egypt; 4 Immunology Department, Theodor-Bilharz Research Institute (TBRI), Giza, 12411 Egypt

## Abstract

Sorafenib (SOR) is the first-line treatment for hepatocellular carcinoma (HCC). However, its use is hindered by the recently expressed safety concerns. One approach for reducing SOR toxicity is to use lower doses in combination with other less toxic agents. Biochanin-A (Bio-A), a promising isoflavone, showed selective toxicity to liver cancer cells. We postulated that combining SOR and Bio-A could be synergistically toxic towards HCC cells. We further evaluated the underlying mechanism. Cytotoxicity assay was performed to determine the IC_50_ of Bio-A and SOR in HepG2, SNU-449 and Huh-7 cells. Then, combination index in HepG2 was evaluated using Calcusyn showing that the concurrent treatment with lower concentrations of SOR and Bio-A synergistically inhibited cell growth. Our combination induced significant arrest in pre-G and G0/G1 cell cycle phases and decrease in cyclin D1 protein level. Concomitantly, SOR/Bio-A reduced Bcl-2/Bax ratio. Furthermore, this co-treatment significantly increased caspase-3 & -9 apoptotic markers, while decreased anti-apoptotic and proliferative markers; survivin and Ki-67, respectively. Active caspase-3 in HepG2, SNU-449 and Huh-7 confirmed our synergism hypothesis. This study introduces a novel combination, where Bio-A synergistically enhanced the anti-proliferative and apoptotic effects of SOR in HCC cells, which could serve as a potential effective regimen for treatment.

Hepatocellular carcinoma (HCC) is a major health problem, accounting for approximately 6% of all human cancers and one million deaths annually^1^,^2^. The incidence of HCC has been rising rapidly in recent decades due to infection with hepatitis C virus (HCV)^3^. HCC patients with a surgically-resectable localized tumor have a much better prognosis, although there is still only a 20–51% 5-year survival rate^4^. Clearly, there is an urgent need for new therapies for this aggressive disease. The antiangiogenic multikinase inhibitor SOR is the first and only systemic agent to notably improve survival in patients with advanced HCC^5^. One limitation of sorafenib (SOR) use is its toxicity^6^. One approach to overcome SOR toxicity is to use lower doses in combination with other complementary agents to potentiate the SOR-mediated tumor inhibition without significant systemic toxicity^7^. Such potentiation could also have cost-benefit advantages due to the high cost of SOR when given at therapeutic doses^8^. Accordingly, natural products with anti-cancer efficacy and low toxicity to normal tissues are suggested as possible candidates to be investigated for their synergistic efficacy in combination with the conventional chemotherapeutic agents^9^. Soy isoflavones are natural chemopreventive agents that are not toxic to normal cells^10^. Bio-A, an isoflavone, is marketed for the alleviation of menopausal symptoms^11^. It also has been reported to have antioxidant^12^, anti-inflammatory^13^, antiviral^14^, and anticarcinogenic effects^15^, and protective effects on endothelial integrity and function^16^. Bio-A has shown to be a promising agent for the treatment of HCC through induction of apoptosis in human hepatoma cells^17^.

In the present study, we aimed to investigate the effect of Bio-A on SOR cytotoxicity in HCC cell lines and investigate the underlying mechanisms for such modulation with emphasis on proliferation and apoptosis in HepG2 cells.

## Results

### Cytotoxic effects of Bio-A and/or SOR on human HCC cell lines

**Figure 1:**
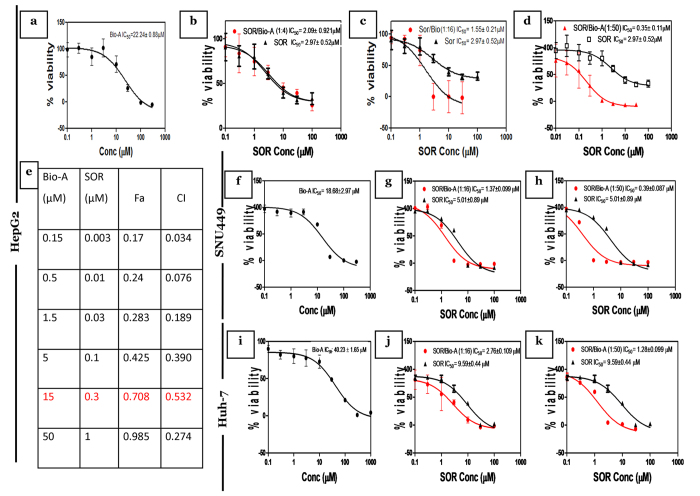
Effect of Bio-A on SOR-mediated cytotoxicity in HCC cells after 72 h treatment. Concentration-response plot of: (**a,f,i**) Bio-A single treatment, (**b**) SOR/Bio-A combination ratio 1:4 in HepG2 cells, (**c,g,j**) 1:16, (**d,h,k**) 1:50 combination ratios in HepG2, SNU 449 and Huh-7, respectively. Data are mean ± SD (n = 6). (**e**) Table showing synergy analysis of 1:50 SOR/Bio-A in HepG2 cells.

To explore the impact of Bio-A on the cytotoxicity of SOR in HepG2 liver cancer cells, concentration-response curves of SOR alone were first assessed and then compared to those obtained after co-treatment with Bio-A for 72 h. Bio-A alone produced inhibition of cell viability with IC_50_ value of 22.24 ± 0.88 μM in HepG2 cell line (Fig. 1a). Combination of SOR/Bio-A at ratios of 1:4, 1:16 and 1:50 showed a decrease in SOR IC_50_ by 30%, 48% and 88%, respectively with the highest reduction in the last and an obvious left shift in the concentration-response curve (Fig. 1b–d). The synergy analysis using Calcusyn software for the 1:4 combination ratio (Supplementary Table 1) showed that the only point showing synergism (<1) had low fraction of affected cells (Fa). Although the 1:16 combination ratio showed synergism with high Fa at two points, the concentration of each agent was high (Supplementary Table 2). Interestingly, the combination ratio 1:50 was found synergistic (<1) at all analyzed concentrations (Fig. 1e) with an increasing Fa from 0.17 till 0.985. Therefore, this combination ratio was used throughout our study and the dose reduction index for this ratio was further analyzed in Supplementary Table 3.

In order to further explore the effect on SNU 449 cells, similar cytotoxicity assays were conducted. Bio-A exhibited IC_50_ value of 18.68 ± 2.97 μM (Fig. 1f), while SOR/Bio-A co-treatment at ratios of 1:16 and 1:50 produced a decrease in SOR IC_50_ by 72.7% and 92.14%, respectively (Fig. 1g,h). Additionally, upon single treatment of Huh-7 cells with Bio-A or SOR, it showed higher IC_50_ values of 40.2 ± 1.23 (Fig. 1i) and 9.59 ± 0.44 μM, respectively compared to the other cell lines. SOR/Bio-A co-treatment at ratios of 1:16 and 1:50 exhibited a decrease in SOR IC_50_ by 71.4% and 87.8%, respectively (Fig. 1j,k).

### Effect of the treatment with combination of SOR and Bio-A on HepG2 cell cycle phases

**Figure 2:**
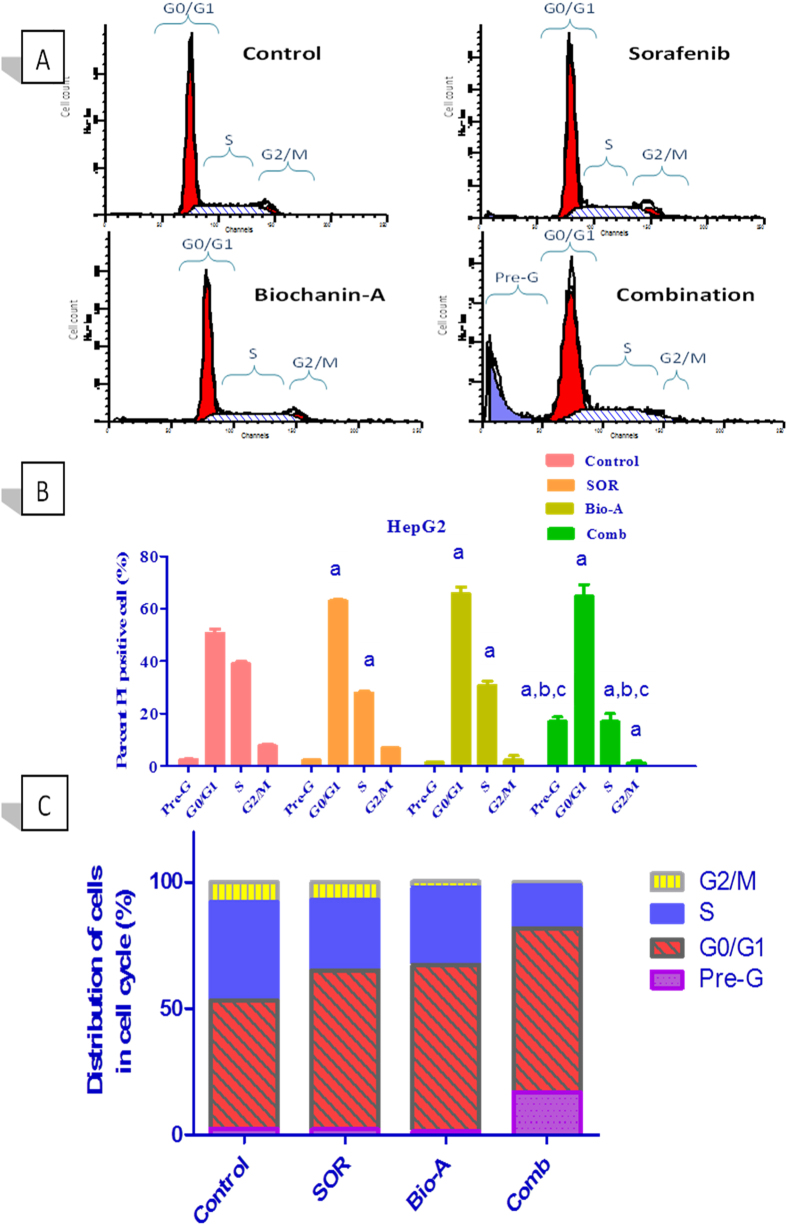
Effect of SOR/Bio-A combination on DNA-ploidy flowcytometric analysis of HepG2 cells treated for 24 h. (**A**) Representative histogram for DNA flowcytometry. (**B**) Bar chart showing effect of treatment on different cell cycle phases. (**C**) Cumulative bar chart. (**a**) significantly different from control; (**b**) significantly different from SOR; (**c**) significantly different from Bio-A. (p < 0.05).

SOR alone induced a significant increase in number of HepG2 cells at G0/G1 phase by 24.28% with significant decrease in S phase by 28.47% compared to control. Number of Bio-A alone treated cells significantly increased at G0/G1 phase by 30.03% while significantly decreased at S phase by 21.13% compared to control. Combined treatment with SOR/Bio-A caused a significant increase in cells accumulated at pre-G and G0/G1 phases by 6.59 and 1.27 (27.92%) folds, respectively compared to control. Additionally, SOR/Bio-A showed significant reduction in HepG2 cells at S and G2/M phases by 1.76 and 6.09 folds compared to control, respectively. SOR/Bio-A co-treatment significantly increased number of arrested cells at pre-G by 7.64 fold and decreased number of cells at S phase by 1.647 fold compared to SOR-alone treated cells (Fig. 2).

### Bio-A enhanced SOR inhibitory effects on cell cycle regulatory protein Cyclin D1

**Figure 3:**
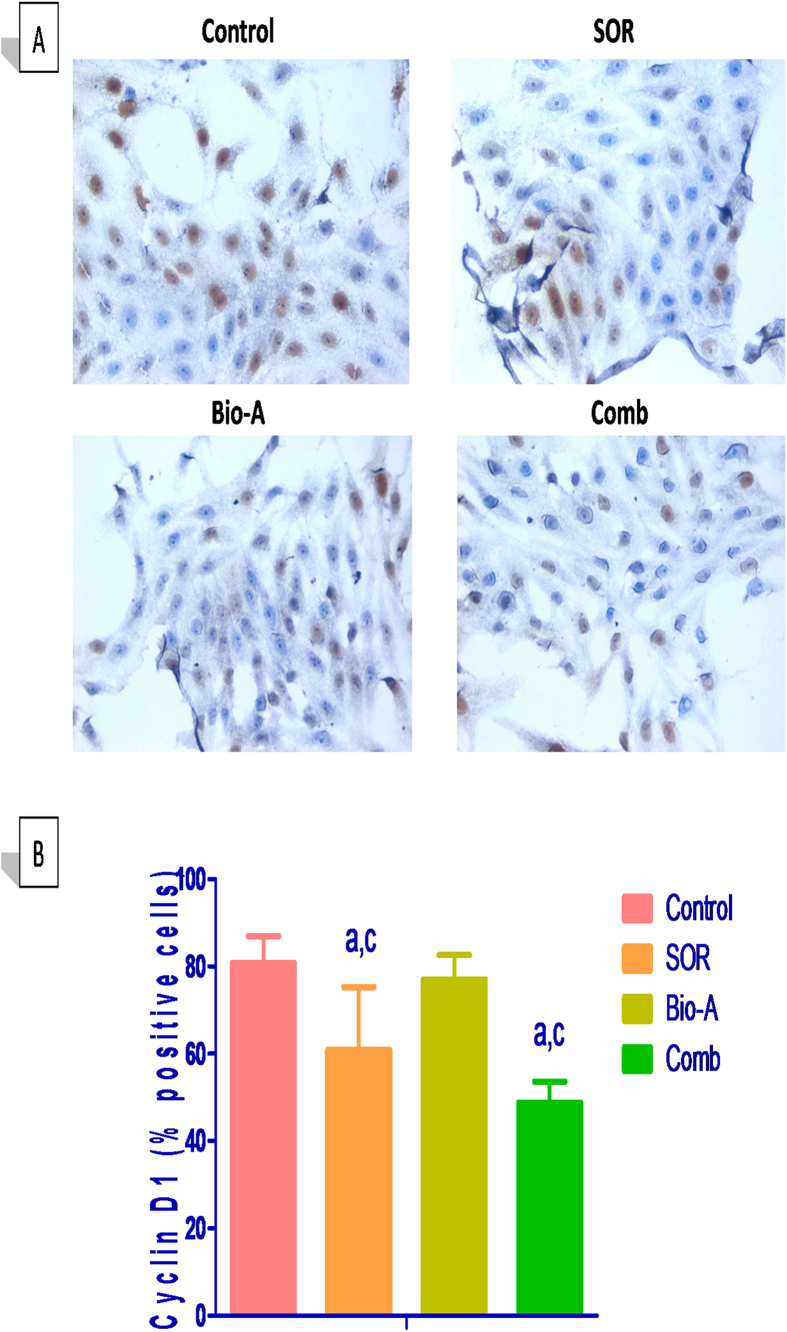
Effect of Bio-A on the protein abundance of cyclin D1 in HepG2 cells treated with SOR for 72 h using immunocytochemistry. (**A**) Representative photomicrograph (ICCX400). (**B**) Bar chart showing cyclin D1 positive cells percentage. (**a**) Significantly different from control; (**c**) significantly different from Bio-A. (p < 0.05).

Co-treatment with SOR/Bio-A showed a significant low-expression in immunocytochemical staining of cyclin D1 protein by 39.5% compared to untreated cells and by 36.6% to Bio-A-alone treated cells. SOR-alone treated cells showed a significant decrease in cyclin D1 abundance by 24.6% and 20.9% compared to untreated cells and Bio-A single treated cells, respectively (Fig. 3).

### Effect of SOR/Bio-A combination treatment on Bax and Bcl-2 intrinsic apoptotic markers

Bax protein expression was significantly increased in SOR/Bio-A co-treated HepG2 cells by 161.26%, 103.011% and 45.68% compared to untreated, Bio-A and SOR single-treated cells, respectively. Also, SOR-alone treated cells significantly showed higher Bax protein expression by 79.33% compared to control.

**Figure 4:**
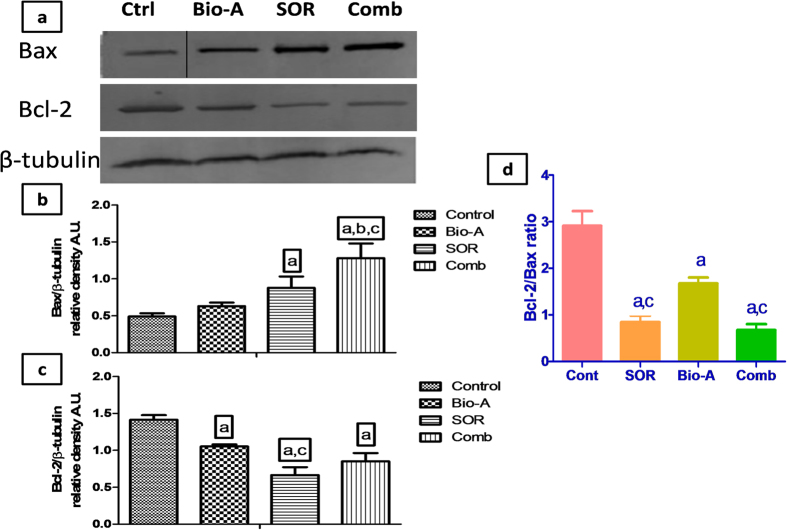
Effect of SOR/Bio-A combination on protein expression of Bax and Bcl-2 in HepG2 cells treated for 72 h. (**a**) Representative western blots, the “Control” band in Bax protein blot was cropped and further represented by another full length replicate blot in Supplementary Figure 1. Bar chart showing: (**b**) Bax protein level, (**c**) Bcl-2 protein level in different groups, (**d**) Bcl-2/Bax ratio. (**a**) significantly different from control; (**b**) significantly different from SOR; (**c**) significantly different from Bio-A. (p < 0.05).

On the other hand, the protein expression of Bcl-2 was significantly decreased in SOR-alone treated cells by 52.98% and 36.81% compared to control and Bio-A treated cells, respectively. SOR/Bio-A combination showed a significant decrease in Bcl-2 expression by 39.90% compared to untreated cells. Bio-A monotherapy induced significant reduction in protein level by 25.58% compared to control (Fig. 4a–c).

Furthermore, Bcl-2/Bax ratio was significantly decreased in SOR-alone treated cells by 71% and 50% compared to control and Bio-A single treated cells, respectively. Bio-A single treatment also significantly decreased Bcl-2/Bax ratio by 42% compared to control. Co-treatment with SOR/Bio-A significantly decreased Bcl-2/Bax ratio by 60% and 77% compared to Bio-A single-treated and untreated cells, respectively (Fig. 4d).

### Effect of combined treatment of SOR and Bio-A on apoptotic marker caspase-9

**Figure 5:**
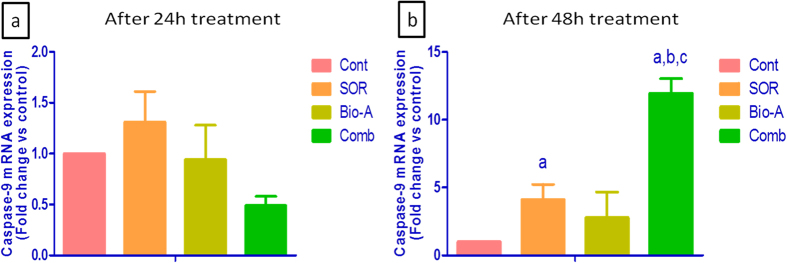
Effect of SOR/Bio-A on the mRNA level of caspase-9 in HepG2 cells. Where (**a**) after 24 h treatment, (**b**) after 48 h treatment. (**a**) Significantly different from control; (**b**) significantly different from SOR; (**c**) significantly different from Bio-A. (p < 0.05).

After 24 h exposure, SOR monotherapy up-regulated mRNA level of caspase-9 in HepG2 cells by 50%, while Bio-A monotherapy increased the level by 18% compared to control. Concomitant SOR/Bio-A treatment down-regulated caspase-9 mRNA by 44.6% compared to control (Fig. 5a).

On the other hand, 48 h exposure to either SOR or Bio-A monotherapy up-regulated the caspase-9 mRNA by 4.104 and 2.777 folds compared to control, respectively. The SOR/Bio-A combination significantly up-regulated the mRNA of caspase-9 by 11.94 and 2.909 folds compared to untreated cells and SOR monotherapy, respectively (Fig. 5b).

### Effect of SOR/Bio-A co-treatment on apoptotic marker caspase-3

**Figure 6:**
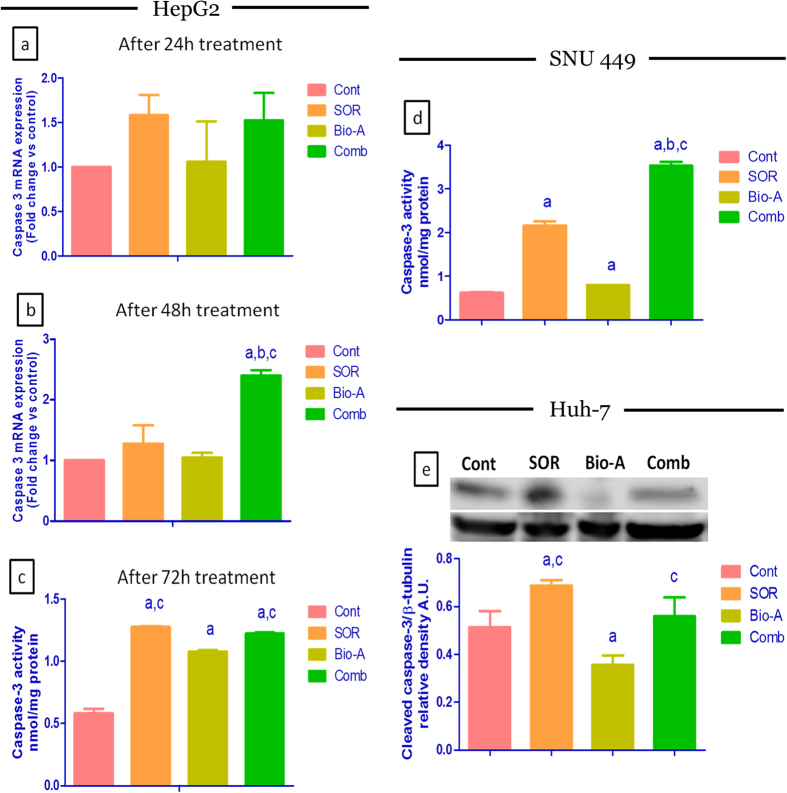
Effect of SOR/Bio-A combination on caspase-3 levels in HCC cells. (**a**) qRT-PCR mRNA levels for 24 h, (**b**) for 48 h treatment in HepG2 cells. (**c**,**d**) Active caspase-3 protein level after 72 h treatment using ELISA in HepG2 and SNU449 cells, respectively. (**e**) Cleaved caspase-3 immunoblot in Huh-7 after 72 h treatment and bar chart showing protein levels in different groups. (**a**) significantly different from control; (**b**) significantly different from SOR; (**c**) significantly different from Bio-A. (p < 0.05).

HepG2 co-treatment with SOR/Bio-A for 24 h (Fig. 6a) up-regulated caspase-3 mRNA level compared to control by 52.5% using qRT-PCR. While, SOR-and Bio-A-alone treated cells up-regulated caspase-3 gene expression by 58.3% and 6%, respectively when compared to untreated cells with no significant difference.

However, after 48 h treatment (Fig. 6b) the caspase-3 mRNA level was significantly up-regulated by 27.3%, 4.5% and 139.9% using SOR, Bio-A monotherapy and the combined therapy compared to control, respectively. SOR/Bio-A combination showed significant caspase-3 mRNA up-regulation by 88.4 and 129.5% compared to SOR and Bio-A monotherapy, respectively.

The effect of HepG2 cells exposure to SOR/Bio-A combination for 72 h on active caspase-3 protein levels (Fig. 6c) was significantly increased by 2.1 and 1.13 folds compared to control and Bio-A single treatment, respectively. SOR single treatment also significantly increased caspase-3 protein level by 2.19 and 1.18 folds compared to control and Bio-A single treatment, respectively. Bio-A single treatment increased caspase-3 protein level significantly by 1.85 fold compared to control.

Additionally, the effect of SOR/Bio-A combination treatment on SNU449 cells was also investigated (Fig. 6d). The active caspase-3 protein levels was significantly increased in SOR and Bio-A single treated groups by 248.2% and 29.2% compared to control, respectively. Interestingly, the SOR/Bio-A co-treatment group showed significant increase by 1.6, 4.4 and 5.6 fold compared to SOR, Bio-A and control groups, respectively.

Furthermore, the effect of SOR/Bio-A co-treatment effect on cleaved caspase-3 protein was examined in Huh-7 (Fig. 6e). The SOR-treated cells showed significant increase in the protein level by 92.8% and 34.2% compared to Bio-A and control groups, respectively. The SOR/Bio-A combination showed a slight non significant decrease by 22.8% compared to SOR-treated cells. While, the co-treatment revealed only significant increase by 57% compared to Bio-A single treated cells.

### Effect of Bio-A addition to SOR on the anti-apoptotic marker survivin

**Figure 7:**
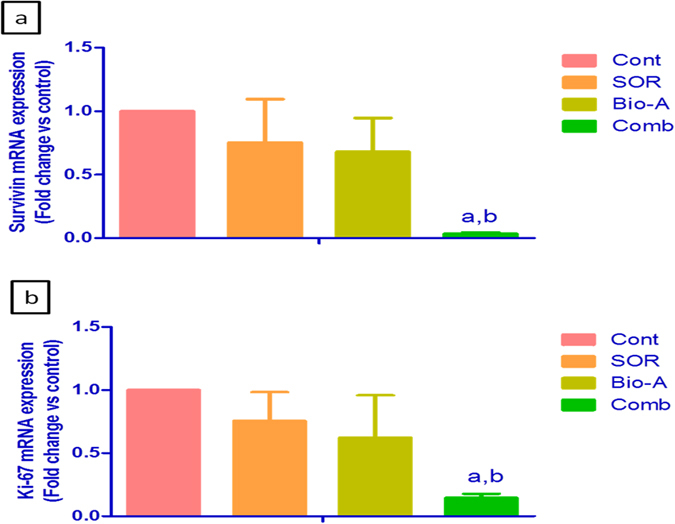
Effect of SOR/Bio-A combination on the gene expression of (**a**) anti-apoptosis survivin. (**b**) Ki-67 proliferation marker in HepG2 cells treated for 48 h. (**a**) significantly different from control; (**b**) significantly different from SOR. (p < 0.05).

Single treatment by either SOR or Bio-A for 48 h down-regulated survivin gene expression by 24.75% and 31.99% compared to control, respectively. Meanwhile, co-treatment with SOR/Bio-A significantly down-regulated survivin gene expression by 96.39% compared to untreated cells and by 95.2% compared to SOR-alone treated cells (Fig. 7a). On the other hand, survivin mRNA levels didn’t show significant changes for 24 h treatment (Data not shown).

### Effect of Bio-A addition to SOR on the proliferation marker Ki-67

Exposure of the cells to either SOR or Bio-A down-regulated Ki-67 mRNA level by 24.41 and 37.67% compared to control, respectively. While, SOR/Bio-A combination showed significant down-regulation in Ki-67 by 85.44% compared to untreated cells and by 80.73% compared to SOR-alone treated cells (Fig. 7b). On the other hand, Ki-67 mRNA showed no significant change after 24 h treatment (Data not shown).

## Discussion

Despite the use of SOR as the first-line treatment for HCC, standard therapeutic regimens for intermediate and advanced staged HCC patients are largely ineffectual resulting in a mean survival time of <12 months after diagnosis^18^. Another limitation is the severity of SOR side effects. Therefore, there is an urgent need to use SOR in combination with less toxic agents, showing potential anti-cancer activity, to approach higher therapeutic efficacy and lower undesirable side effects. Bio-A was recently reported to increase the sensitivity of pancreatic cancer cells to atorvastatin as an anti-proliferative agent^19^. Moreover, genistein, a major soy isoflavone and a Bio-A metabolite, was found to synergize SOR cytotoxic effects in malignant neuroblastoma cells^20^. So far, there have been no reports on the incorporation of Bio-A in combination with SOR. Therefore, we investigated in the current study whether our combination has synergistic antitumor activity in human HCC cells while using lower SOR dose than SOR alone with high dose.

Our data showed that Bio-A exhibited cytotoxic potential towards HepG2 and Huh-7 cells with IC_50_ 22 and 40 μM, respectively. This finding is in line with previous published data about the cytotoxic potential of Bio-A in human hepatoma cell lines^17^. To the best of our knowledge this is the first study to search the anticancer potential of Bio-A on SNU 449. By assessing the concentration-response curves for SRB cytotoxicty assay of the different combination ratios in HepG2, there was a significant left shift in the 1:50 combination ratio compared to SOR-alone concentration response curve. Afterwards, combination index and dose reduction index values for the combination of 0.3 μM SOR and 15 μM Bio-A indicated that Bio-A synergized the cytotoxicity of SOR in HepG2 at 1:50 combination ratio while minimizing the concentrations of both agents and this combination was used throughout our study. Interestingly, the 1:50 combination ratio showed the same pattern in SNU 449 with pronounced SOR concentration reduction. On the contrary, SOR dose reduction was slightly hampered in Huh-7 which could be associated with the high initial SOR and Bio-A IC_50_. The high IC_50_ in Huh-7 could be attributed to either the cells’ advanced stage of HCC or the sensitivity of WST-1 in comparison with SRB cytotoxicity assays.

The control of the cell cycle is one of the principal tasks of the cell. Although the process is routine, the cell makes a decision at every nanosecond about its fate that can compromise normal replication, apoptosis, necrosis, or uncontrolled growth that can finally lead to cancer development^21^. Therefore, interference with coordinated cell cycle progression induces apoptosis^22^. In the present study, DNA flowcytometry indicated that monotherapy by either SOR or Bio-A alone induced cell cycle arrest at G0/G1 together with a significant decrease in S-phase. Moreover, SOR was previously reported to induce G1-phase arrest and reduce the S-phase subpopulation in HCC cells^23^, which further supports our findings. In a separate study, SOR was found to paradoxically increase accumulation of cells at S/G2/M phases^24^. On the other hand, Su *et al*.^17^ reported that Bio-A did not induce cell cycle arrest in HCC cells. Therefore, we introduce for the first time the effect of Bio-A on HepG2 cells, and this could be related to the work of Rice *et al*.^25^ who indicated that Bio-A induced cell cycle arrest in prostatic carcinoma LNCap cells at G0/G1 phase. Herein, cell cycle analysis revealed significant accumulation at pre-G and G0/G1 for SOR/Bio-A combination, which indicated synergistic cell cycle arrest and enhanced cell death. This shows the augmented modulatory effect of Bio-A on cell cycle distribution when combined with SOR at one-tenth of the original SOR IC_50_ on HepG2 cells.

Additionally, results of the cell cycle analysis were further supported by assessing the abundance of the cell cycle regulatory protein cyclin D1, an important regulator of G1-to-S phase progression^26^. Moreover, cyclin D1 is considered to play a role in promoting the process of apoptosis^27^. Our results showed that cyclin D1 is down-regulated in HepG2 cells treated with SOR, which is consistent with those of Fernando *et al*.^24^ and Deng *et al*.^23^. Despite that Bio-A alone has no significant impact on cyclin D1 protein level, it potentiated SOR effect when they were used in combination at reduced SOR IC_50_.

Apoptosis, also known as ‘programmed cell death’, is a phenomenon of the body to eliminate the unwanted and cancerous cells. The ability of internal or external stimuli to induce cell death is, therefore, recognized for its immense therapeutic potential^28^. Mechanistically, tumor inhibition by combination therapies may result from an increased capacity of these drugs to induce apoptosis. Therefore, the present study investigated the impact of combining Bio-A with SOR on mitochondrial apoptosis signaling pathway to elucidate the underlying mechanism behind the synergistic interaction. Bcl-2 (B-cell lymphoma 2) and Bax (Bcl-2 associated X protein) are known to play critical roles in apoptosis, and most interestingly the ratio of Bcl-2 and Bax reflects the status of apoptosis^29^. Bax is one of the proapoptotic proteins which promote the process of apoptosis^30^ and was proven to be expressed at low levels in human hepatoma cells^31^. In this study, immuno-blotting analysis indicated a significant increase in Bax protein expression after 72 h exposure of the cells to SOR-only treated cells when compared to control. This finding gains support by the work of Fernando *et al*.^24^. Indeed, so far there has been no published data showing the impact of Bio-A on Bax protein level in HepG2 cells except the work of Su *et al*.^17^ who reported that Bax protein levels were undetectable after 24 h exposure to Bio-A. The increase in Bax protein level in the present work after exposure to Bio-A showed no significant difference when compared to untreated cells. Meanwhile, the addition of Bio-A to SOR lead to significant over-expression of Bax protein compared to SOR-alone treated HepG2 cells. Bcl-2 is another important regulator of mitochondrial apoptosis^32^. A study in Bcl-2 transgenic mice has shown that ectopic expression of Bcl-2 in the liver can protect normal hepatocytes from apoptosis^33^. Loss of Bcl-2 expression can induce apoptosis in cancer cells^34^. Immunohistochemical studies have shown that Bcl-2 is not generally expressed in human hepatocytes^35^, whereas Su *et al*.^17^ showed that Bcl-2 was expressed in three human hepatoma cell lines including HepG2. Throughout the current work, SOR treatment produced a significant decrease in Bcl-2 protein level compared to untreated HCC cells. This is in concordance with the work reported by Zhang *et al*.^36^ and Sonntag *et al*.^37^. Bio-A single treatment also reduced Bcl-2 protein level compared to control untreated cells. This finding is in consonance with the effect of Bio-A on Bcl-2 in HepG2 cells showed by Su *et al*.^17^. Additionally, Concomitant SOR/Bio-A treatment resulted in a significant reduction in Bcl-2 protein when compared to control group. A decrease in Bcl-2/Bax ratio was observed with SOR/Bio-A co-treatment compared to control and Bio-A single treated cells. This ratio determines whether a cell responds to an apoptotic signal^38^. This further supports that SOR/Bio-A combination has a favorable synergistic effect towards induction of apoptosis at least partly through the mitochondrial pathway. Since caspases are reported to be the main executors of the apoptotic process by carrying out the execution of cellular demolition^39^. Our next step was to assess caspase-9. Caspase-9 is an initiator caspase downstream to mitochondrial apoptosis pathway^40^. Our results showed no significant change in caspase-9 mRNA after 24 h exposure. While, after 48 h SOR-alone treated group showed significant increase in caspase-9 mRNA level. This is in agreement with the work of Chen *et al*.^41^. Nevertheless, SOR/Bio-A combination significantly increased Caspase-9 gene expression compared to SOR-alone and control groups. These results showed that the change in caspase-9 levels is time-dependant. This could be related to mitochondrial-mediated apoptotic pathway activation which results in caspase-9 activation^42^. Activated caspase-9 activates executioner caspases, including caspase-3, which in turn cleave a number of cellular proteins that include structural proteins, nuclear proteins, cytoskeletal proteins, and signaling molecules^40^. Therefore, the effect of the different treatments on caspase-3, a crucial hallmark in the apoptosis signaling pathway, was assessed. Our study revealed that change in caspase-3 levels in HepG2 cells were time-dependant. In alignment with caspase-9, there were no significant changes in caspase-3 mRNA levels after 24 h treatments. However, treatment with SOR and Bio-A for 48 h synergistically upregulated caspase-3 mRNA level compared to SOR-alone and control groups. Moreover, SOR/Bio-A combination elevated active caspase-3 protein level after 72 h exposure compared to untreated cells. Since the activation of effector caspases as Caspase-3 requires the activation of initiator caspases, such as caspase-9, in response to pro-apoptotic signals^43^. Caspase-3 results could be linked with caspase-9 time-dependant changes with SOR/Bio-A treatment. In a similar manner, active caspase-3 level was elevated in SNU 449 cells after 72 h exposure to SOR/Bio-A combination treatment. In order to examine the effect of the same combination ratio and concentrations used in the former two cell lines, we explored the effect of the tremendous reduction in the SOR concentration and using only 3% of the initial IC_50_ in combination with 37% of Bio-A IC_50_ in Huh-7 cells. Interestingly, the observed results showing no significant reduction in cleaved caspase-3 protein level compared to SOR-treated cells at the normal IC_50_ and significant increase in the protein level compared to Bio-A treated cells, supports our previously discussed results and hypothesis despite using very low concentrations.

Survivin is an inhibitor of apoptosis protein^44^. It is known to bind with microtubules of the mitotic spindle^45^ and inhibiting caspase activity^46^. Although, Cervello *et al*.^47^ showed a reduction in survivin protein level after SOR treatment in HepG2 cells, neither SOR nor Bio-A individual treatments displayed significant downregulation in survivin mRNA level in the current study. However, SOR/Bio-A combination exhibited significant down-regulation in survivin mRNA level. This could support the evidenced up-regulation in caspase-9 and caspase-3 level in cells co-treated with SOR and Bio-A. Survivin is also known to control proliferation through affecting G1 checkpoint^48^. This can further support DNA cell cycle analysis results showing G0/G1 arrest upon co-treatment with SOR and Bio-A.

The anti-proliferative effect of SOR/Bio-A combination was further verified by assessing the level of mRNA transcripts of the proliferation marker Ki-67. This marker is strongly correlated to a neoplastic proliferative status and activity. It is expressed at higher levels in the nuclei of proliferating cells but it is absent in resting ones^49^. qRT-PCR results revealed that treatment with SOR and Bio-A combination significantly down-regulated Ki-67 gene expression, which shows synergistic anti-proliferative activity towards HepG2 cells. However, there was no significant decrease in Ki-67 mRNA level neither by SOR nor Bio-A single treatments. On the contrary, a previous study stated that SOR down-regulated Ki-67 in HCC mouse xenograft model^50^.

**Figure 8:**
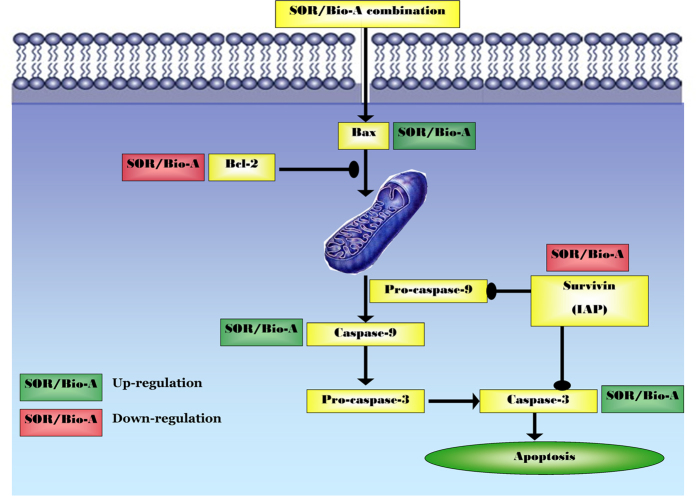
Schematic diagram of the probable mechanism of synergy between SOR and Bio-A through mitochondrial apoptosis signaling pathway.

In summary, the present study provided evidence that the combination of SOR and Bio-A leads to synergistic cytotoxicity in the HCC cell line, HepG2, *via* interfering with cell cycle, enhancing mitochondrial apoptosis signaling and thus hindering cell proliferation as summarized in Fig. 8. In addition, using the same ratio and concentration of the SOR/Bio-A combination in SNU 449 and Huh-7, showed decreased cell proliferation and enhanced caspase-3 activity. This warrants further *in-vivo* studies in animal xenograft models.

## Experimental procedures

### Cell Culture

Hepatoma cell lines; HepG2, SNU449 and Huh-7 were obtained from the American Type Culture Collection (ATCC, Manassas, VA, USA, were cultured in RPMI1640 supplemented with 100 μg/mL streptomycin, 100 units/mL penicillin and 10% heat-inactivated fetal bovine serum (Lonza, Basel, Switzerland) at 37 °C in a humidified, 5% (v/v) CO_2_ atmosphere. Cells were serially passaged at 80–90% confluency.

### Experimental Design

Hepatoma cells were divided into four groups: the blank control group, cells were treated with the vehicle; SOR group, cells were treated with SOR IC_50_ (Cayman chemical, Ann Arbor, MI, USA); Bio-A group, cells were treated with biochanin-A IC_50_ (Sigma-Aldrich Co., St. Louis, MO, USA); Bio-A + SOR combination group, cells were treated with 0.3 μM SOR and 15 μM biochanin-A. All treatments were started 24 hrs after cells were seeded in either 96-well plates or T-25 flasks. mRNA levels were tested either at 24 h or 48 h treatment exposure, while protein expression levels were done at 72 h exposure.

### Cytotoxicity Assay and Synergy Analysis

SOR and Bio-A were dissolved in DMSO (Sigma-Aldrich Co., St. Louis, MO, USA) and kept at a stock concentration of 100 mM. Initially, single-drug concentration–effect curves were assessed. Seeding was done at a density of 3,000 cells/well in 96-well plates. Cells were exposed to different treatments for 72 h during which ten different drug concentrations were tested. Bio-A was used at concentrations of 0.1–1000 μM, while SOR was used at concentrations of 0.01–100 μM. Cytotoxicity was assessed at the end of drug exposure for HepG2 and SNU449 using SRB assay as described previously^51^. Absorbance was measured at 545 nm using a microplate reader (ChroMate-4300, FL, USA). On the other hand, WST-1 cytotoxicity assay was done on Huh-7. Results were expressed as the relative percentage of absorbance compared to control. Experimental conditions were tested using six replicates (six wells of the 96-well plate per experimental condition) and all experiments were performed in triplicates. Half-maximal inhibitory concentration (IC50), the drug concentration at which 50% growth inhibition is achieved, was calculated using GraphPad Prism software, version 5.00 (GraphPad Software, Inc. La Jolla, CA, USA). Drug interactions were analyzed by CalcuSyn program, Version 2.1 (Biosoft, Cambridge, UK) based on the analytical method of Chou and Talalay^52^.

### DNA-Flow Cytometry Analysis

HepG2 cells were seeded at a density of 7 × 10^5^ cells in T-25 flasks for 24 h and then were exposed to different treatments for another 24 h. The cells were collected by trypsinization, washed in PBS and then fixed in ice-cold absolute alcohol. Thereafter, cells were stained using CycleTEST™ PLUS DNA Reagent Kit (BD Biosciences, San Jose, CA according to the manufacturer’s instructions. Cell-cycle distribution was determined using a FACSCalibur flow cytometer (BD Biosciences, San Jose, CA).

### Immunocytochemistry

HepG2 cells were seeded on charged slides (Menzel Gläser, Braunschweig, Germany), pre-coated with sterile-filtered poly L-lysine, at a density of 2 × 10^6^ cells/100 mm dish. After exposure to different treatments for 72 h, cells were fixed with absolute ethanol, washed in phosphate buffered saline (PBS) and incubated with 0.01% Triton X-100 in PBS for 1 min to permeabilize the cell membranes. Cells were afterwards incubated with freshly prepared 10% H_2_O_2_ in mehanol for 30 min to quench endogenous peroxidase activity and then washed with PBS (pH 7.4). To block the non-specific binding of the secondary antibody, cells were incubated in 5% normal horse serum in Tris-buffered saline plus Tween-20 (TBST) for 30 min. Thereafter, cells were incubated overnight in covered humid chamber at 4 °C with primary cyclin D1 rabbit polyclonal antibody (1:50 dilution, Thermo Fisher Scientific, UK). In the following day, the slides were incubated with the corresponding conjugated anti-rabbit IgG (1:2000 dilution, Santa Cruz Biotechnology Inc., Dallas, TX, USA). Cells were treated afterwards with streptavidin horseradish peroxidase complex (1:100 dilution, ABC/HRP; Vector Laboratories, Burlingame, CA, USA) in TBST for 50 min. The color reaction was developed for 5 min in 3,3′-diaminobenzidine (DAB) solution (Santa Cruz Biotechnology Inc., Dallas, TX, USA). Percentage of DAB-positive cells per high power field was calculated using imageJ software (National Institutes of Health, Bethesda, Maryland).

### Quantitative Real-Time Polymerase Chain Reaction (qRT-PCR)

Total RNA isolation from cell pellet was performed using RNeasy Mini Kit® (Qiagen Inc., Valencia, CA, USA). RNA was then quantified using Nanodrop 2000 (Thermo Scientific, Wilmington, USA) and reverse transcription was performed using a total of 1 μg of the RNA, in a total reaction of 20 μl, using the High Capacity cDNA Reverse Transcription kit (Applied Biosystems, Foster City, CA, USA) using the manufacturer’s protocol. The produced cDNA is then used as a template, from which 2 μl were used for real-time PCR reaction. Cycling conditions were as follows; initial denaturation step at 95 °C for 10 min, followed by 45 cycles of; 95 °C for 15 sec and annealing temperature 60 °C for 1 min, and a final extension step at 60 °C for 10 min. For endogenous control, β-actin was used in each experiment as the housekeeping gene with forward primer GGC-ATC-CTG-ACC-CTG-AAG-TA and reverse primer GGG-GTG-TTG-AAG-GTC-TCA-AA. Relative expression was calculated using the comparative C_t_ method. Expression of the gene of interest was calculated as fold induction compared with control group and was corrected with the quantified expression level of β-actin. Quantitative real-time PCR analysis was performed using the 7500 Real-Time PCR System (Applied Biosystems). Primer sequences were as follows: Caspase-3 forward primer TTC-AGA-GGG-GAT-CGT-TGT-AGA-AGT-C and reverse primer CAA-GCT-TGT-CGG-CAT-ACT-GTT-TCA-G; caspase-9 forward primer GCG-AAC-TAA-CAG-GCA-AGC-AG and reverse primer ACC-ACG-CAG-CAG-TCC-AGA-GC; survivin forward primer TGC-CCC-GAC-GTT-GCC and reverse primer CAG-TTC-TTG-AAT-GTA-GAG-ATG-CGG-T; ki-67 forward primer CTT-TGG-GTG-CGA-CTT-GAC-G and reverse primer GTC-GAC-CCC-GCT-CCT-TTT.

### Western Blot Analysis

Cells were seeded, cultured and exposed with different treatments for 72 h. Whole cell protein lysates were prepared according to standard protocol using RIPA buffer (0.5 M Tris–HCl, pH 7.4, 1.5 M NaCl, 2.5% deoxycholic acid, 10% NP-40, 10 mM EDTA). Protein (50 μg) was loaded per well of a 12% SDS PAGE gel using electrophoresis buffer (0.192 M glycine, 25 mM Tris, 0.1% SDS). After electrophoresis, the gel was transferred onto a PVDF membrane (Bio-Rad Laboratories, Inc., CA, USA) using transfer buffer (0.192 M glycine, 25 mM Tris, 0.025% SDS, 10% methanol). Membranes were blocked in TBS-T with 5% non-fat dry milk and incubated overnight with the primary anti-Bax (1:1000, Abcam), anti-Bcl-2 antibody (1:250, Abcam) or anti-cleaved-caspase-3 (1:1000, Cell signaling), then incubated with secondary HRP-linked antibody (1:5000) (KPL Inc., USA). Detection was done by Abcam Optiblot ECL Detect Kit (Abcam, USA). Anti-β-tubulin antibody (1:20000) (Sigma-Aldrich Co., St. Louis, MO, USA) was used to for loading correction.

### ELISA Immunoassay

The level of active caspase-3 was assessed using ELISA colorimetric kit as described in Eldehna *et al*.^53^.

### Statistical Analysis

Data are presented as mean ± SD. Comparisons were carried out using one-way analysis of variance (ANOVA) followed by Tukey– Kramer’s test for *post hoc* analysis. To compare the effect of different groups on cell cycle phases, two-way ANOVA was used followed by Bonferroni’s test for *pot hoc* analysis. Statistical significance was acceptable to a level of P < 0.05. All statistical analyses were performed and plotted using GraphPad Prism software, version 5.00 (GraphPad Software, La Jolla, CA).

## Additional Information

**How to cite this article**: Youssef, M. M. *et al*. Novel combination of sorafenib and biochanin-A synergistically enhances the anti-proliferative and pro-apoptotic effects on hepatocellular carcinoma cells. *Sci. Rep.* **6**, 30717; doi: 10.1038/srep30717 (2016).

## Supplementary Material

Supplementary Information
