# Supplementary material for: Novel combination of sorafenib and biochanin-A synergistically enhances the anti-proliferative and pro-apoptotic effects on hepatocellular carcinoma cells
Source: Sci Rep. 2016 Jul 29;6:30717. doi: 10.1038/srep30717 (PMC4965826; doi:10.1038/srep30717)

Novel combination of sorafenib and biochanin-A synergistically enhances the anti-proliferative and pro-apoptotic effects on hepatocellular carcinoma cells

Mohieldin M. Youssef [1,2] , Mai F. Tolba [3], Eman El-Ahwany [4], Amani E. Khalifa [3], Suher Zada [1] and Ashraf B. Abdel-Naim[3]*

[1] The American University in Cairo, New Cairo, Egypt 11835

[2] Pharmacology and Toxicology department, Faculty of Pharmacy, Egyptian-Russian University, Cairo, Egypt 11829

[3] Pharmacology and Toxicology department, Faculty of Pharmacy, Ain-Shams University, Cairo, Egypt 11566

[4] Immunology Department, Theodor-Bilharz Research Institute (TBRI), Giza, Egypt 12411

**Corresponding Author:*

Dr. Ashraf B. Abdel-Naim

Professor of Pharmacology and Toxicology

Faculty of Pharmacy, Ain-Shams University.

Cairo, Egypt 11835

E-mail: abnaim@pharma.asu.edu.eg
Cell: +2 01119998505

Supplementary Table 1: Synergy analysis of SOR/Bio-A combination ratio 1:4 in HepG2 cancer cell line using Calcusyn software.

Bio-A (µM)	SOR (µM)	Fa	CI
0.04	0.01	0.07	0.550
0.12	0.03	0.04	3.756
0.4	0.1	0.092	3.647
1.2	0.3	0.164	4.401
4	1	0.254	6.922
12	3	0.377	9.629
40	10	0.546	13.281
120	30	0.607	29.307
400	100	0.745	46.983

Supplementary Table 2: Synergy analysis of SOR/Bio-A combination ratio 1:16 in HepG2 cancer cell line using Calcusyn software.

Bio-A (µM)	SOR (µM)	Fa	CI
16	1	0.259	7.552
48	3	0.993	0.442
160	10	0.998	0.836
480	30	0.999	1.041

Supplementary Table 3: Dose reduction index (DRI) for SOR/Bio-A 1:50 combination ratio in HepG2 cancer cell line using Calcusyn software.

	Drug alone		Dose Reduction Index (DRI)	
Fa	Bio-A (µM)	SOR (µM)	Bio-A	SOR
0.17	8.0602	0.1994	53.735	66.468
0.24	10.1888	0.3706	20.378	37.058
0.283	11.4964	0.5100	7.664	16.999
0.425	16.1452	1.2518	3.229	12.518
0.708	30.7128	6.8558	2.048	22.853
0.985	183.1680	770.6528	3.663	770.653

Supplementary Figure 1: Representative western blot for the effect of SOR/Bio-A combination on protein expression of Bax in HepG2 cells treated for 72 h

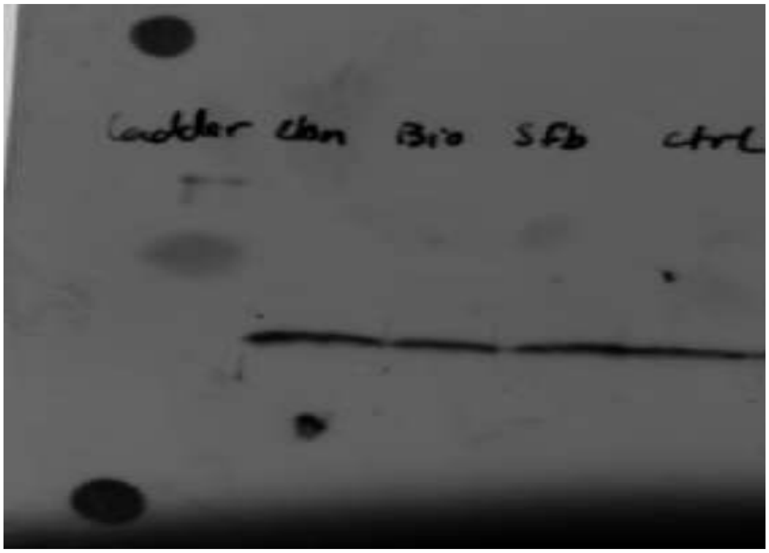

Supplement: Supplementary Information [file srep30717-s1.pdf]
